# Plantar Foot Microvascular Reactivity and Oxygenation in Type 2 Diabetes and Peripheral Artery Disease: An Exploratory Study

**DOI:** 10.1111/micc.70078

**Published:** 2026-07-29

**Authors:** Josi Gallo, Allison Miller, Emily Chan, Grace Chen, Luke Benner, Erik Sillaste, Brhandom Brisueno Lopez, Carolyn Bakx, Bohyun Ro, Juan Orsi, Lara Soares de Araujo, Ernesto Ramirez, Eloisa Herrera Ospina, Qifan Song, Daniel M. Hirai, Igor A. Fernandes, Bruno T. Roseguini

**Affiliations:** ^1^ John Martinson Honors College Purdue University West Lafayette Indiana USA; ^2^ Department of Health and Kinesiology Purdue University West Lafayette Indiana USA; ^3^ Indiana University School of Medicine West Lafayette West Lafayette Indiana USA; ^4^ Laboratory of Applied Sport Physiology University of Campinas Limeira São Paulo Brazil; ^5^ Department of Statistics Purdue University West Lafayette Indiana USA

**Keywords:** heat therapy, peripheral artery disease, plantar microcirculation, type 2 diabetes

## Abstract

**Objective:**

Microvascular dysfunction contributes to foot complications in type 2 diabetes (T2D) and peripheral artery disease (PAD), yet has been characterized primarily in non‐plantar regions. We evaluated microvascular reactivity and oxygenation at ulcer‐prone plantar sites and responses to combined heat therapy and intermittent pneumatic compression (HT + IPC).

**Methods:**

Forty adults ≥ 50 years (Control *n* = 20, T2D *n* = 11, PAD with or without T2D *n* = 9) were studied. Plantar hallux CVC (laser Doppler flux/MAP) and forefoot StO_2_ (near‐infrared spectroscopy) were measured during local heating and reactive hyperemia. Responses to 60‐min HT + IPC were evaluated using the contralateral foot as a control.

**Results:**

At the plantar hallux, the increase in %CVCmax from baseline to the initial peak was attenuated in PAD compared with controls and T2D (*p* < 0.01), whereas plateau responses were similar. At the forefoot, PAD showed faster deoxygenation during ischemia and slower reoxygenation during reactive hyperemia (*p* < 0.01). During HT + IPC, popliteal blood flow increased similarly across groups; however, plantar StO_2_ increased in controls and T2D but remained below baseline in PAD (*p* < 0.001).

**Conclusions:**

These preliminary findings indicate impaired plantar microvascular function in a heterogeneous PAD cohort, most of whom also had T2D. Larger studies are needed to confirm these findings and to elucidate the underlying mechanisms.

## 1 Introduction

Individuals with type 2 diabetes (T2D) and peripheral artery disease (PAD) experience a broad spectrum of foot complications, including sensory, autonomic, and motor dysfunctions that substantially compromise tissue health and function [1]. Among these complications, diabetic foot ulcers (DFUs) represent one of the most serious, disabling, and costly sequelae, affecting more than 18 million individuals worldwide [2]. DFUs most commonly develop on the plantar surface of the toes and forefoot, where repeated mechanical loading and impaired tissue perfusion converge [3, 4, 5]. The presence of DFUs is associated with profound reductions in quality of life and markedly increased risks of hospitalization, lower‐extremity amputation, and mortality [6, 7, 8, 9]. Major risk factors for DFU development include peripheral neuropathy, PAD, and structural foot deformities [2]. Once established, ulcer healing is often prolonged, frequently requiring several months to more than a year, depending on wound severity and patient characteristics [5, 10]. Current standards of care, such as wound debridement, pressure offloading, and specialized dressings, are costly, variably accessible, and often insufficient, with many ulcers failing to heal despite optimal management [2, 11]. Moreover, recurrence rates remain unacceptably high, with approximately 42% of patients developing a recurrent DFU within 1 year and 65% within 5 years of healing [6, 12]. Collectively, these challenges underscore an urgent need not only for effective preventive and therapeutic interventions but also for physiological assessments that can identify ulcer‐prone regions and tissue vulnerability in high‐risk individuals.

The pathogenesis of DFUs is multifactorial [2], but early microcirculatory impairments in the foot skin and skeletal muscle are thought to play a central role [13]. Cutaneous microvascular dysfunction, typically manifested as attenuated vasodilatory responses to local heating or iontophoresis of vasoactive agents, has been extensively documented in individuals with or at risk for T2D [14, 15, 16, 17, 18, 19, 20, 21, 22, 23, 24]. Similar abnormalities are observed in PAD, where patients exhibit blunted vasodilatory responses to thermal and other stimuli in the lower extremities [25, 26, 27, 28]. These alterations are not limited to the skin but extend to the underlying musculature, where impaired oxygenation during contractions and reduced cellular energy reserves have been demonstrated [29, 30]. In more advanced stages of PAD, tissue oxygenation in the foot is markedly reduced, particularly in regions adjacent to chronic wounds [31, 32]. Intriguingly, most previous studies evaluating microvascular dysfunction in populations at risk for DFUs have focused on the dorsal foot rather than the plantar, weight‐bearing regions where ulcers most commonly develop [4]. This distinction is physiologically important, as the plantar skin is structurally and functionally specialized compared with the dorsal surface [33, 34]. In particular, arteriovenous anastomoses are far more abundant in glabrous skin of the hands and feet, including the plantar surface and digital nail beds, whereas they are sparse or absent in non‐glabrous skin, such as the dorsal foot [34]. These specialized vessels are characterized by large diameters, thick muscular walls, and dense sympathetic innervation [34]. In addition, the plantar surface is exposed to repetitive mechanical loading and is more susceptible to neuropathic complications in individuals with diabetes [2, 5]. Characterizing microvascular function in these ulcer‐prone plantar regions may therefore provide important insights into the mechanisms underlying the initiation and progression of DFUs.

Heat therapy (HT) is a simple, practical approach that can increase blood flow and potentially restore microvascular function in individuals at high risk for DFUs [35]. Immersion of the legs in warm water significantly increases limb blood flow, thereby improving vascular function in older adults [36, 37]. In patients with PAD, lower‐body heating with warm water results in a nearly threefold increase in popliteal artery blood flow [38]. Despite these benefits, repeated water immersion is contraindicated for individuals with active ulcers [39, 40], limiting its clinical utility. As an alternative, portable water‐circulating garments provide controlled local heating without submersion and are safe for home use [41, 42, 43, 44, 45]. We previously demonstrated that a single 90‐min session of heating using tube‐lined, water‐circulating trousers doubled popliteal artery blood flow in patients with PAD [44]. We have also developed a novel leg‐sleeve device that combines local heating with low‐grade intermittent pneumatic compression (IPC) to enhance heat transfer and limb perfusion [46]. However, whether HT‐induced increases in bulk leg blood flow translate into improved oxygenation of the plantar foot, the region most vulnerable to ulceration [3, 47], remains unknown. If effective, such garments may represent a feasible strategy for targeting distal microvascular dysfunction and reducing DFU risk.

The present exploratory study had two main goals. First, we compared cutaneous microvascular function at the plantar hallux and plantar forefoot tissue oxygenation among individuals with T2D, individuals with PAD with or without T2D, and control participants without T2D or PAD. Second, we assessed the feasibility and acute physiological effects of combined HT and low‐pressure IPC delivered through a boot‐like garment applied to the lower leg and foot. We evaluated changes in foot temperature, plantar forefoot oxygenation, and leg blood flow during a single 60‐min session. We hypothesized that participants with T2D and PAD would exhibit attenuated plantar microvascular responses compared with controls, with the most pronounced impairments observed in those with PAD, owing to their elevated risk, ulceration, and impaired healing [11]. We further hypothesized that the boot‐like device would improve foot oxygenation and leg blood flow across all groups, but that these responses would be blunted in individuals with T2D and especially in those with PAD.

## 2 Methods

### 2.1 Study Overview

This exploratory study was performed at Purdue University in West Lafayette, Indiana. The protocol was approved by Purdue's Institutional Review Board (IRB‐2025‐195). Written informed consent was obtained from all participants, and all procedures complied with the U.S. Federal Policy for the Protection of Human Subjects (45 CFR Part 46) and were conducted in accordance with the ethical principles of the Declaration of Helsinki. The first participant was enrolled on May 19, 2025, and the final assessment occurred on November 6, 2025.

### 2.2 Participants

Participants were recruited through campus advertisements, an online newsletter, a recruitment agency (Trial Facts), and referrals from local endocrinology and vascular surgery clinics. Three cohorts were enrolled: (1) adults with type 2 diabetes (T2D) without peripheral artery disease (PAD), (2) adults with PAD with or without T2D, and (3) age‐matched controls without T2D or PAD. All participants were men or women aged ≥ 50 years.

Participants were assigned to study groups according to glycemic status and ankle–brachial index (ABI) controls had no clinical diagnosis of diabetes, HbA1c < 6.5%, and ABI > 0.90 in both legs; T2D participants had a clinical diagnosis of type 2 diabetes, HbA1c ≥ 6.5%, and ABI > 0.90; PAD participants had ABI ≤ 0.90 in at least one leg, irrespective of diabetes status. Exclusion criteria included active cancer, impaired thermal sensation in the lower extremities, open ulcers or active skin infection of the feet or legs, dialysis‐dependent kidney disease, body mass index > 40 kg/m^2^, insulin‐dependent type 1 diabetes, recent major cardiovascular events, critical limb ischemia, recent lower‐extremity revascularization or orthopedic surgery, and prior major lower‐limb amputation.

### 2.3 Experimental Protocol

**FIGURE 1:**
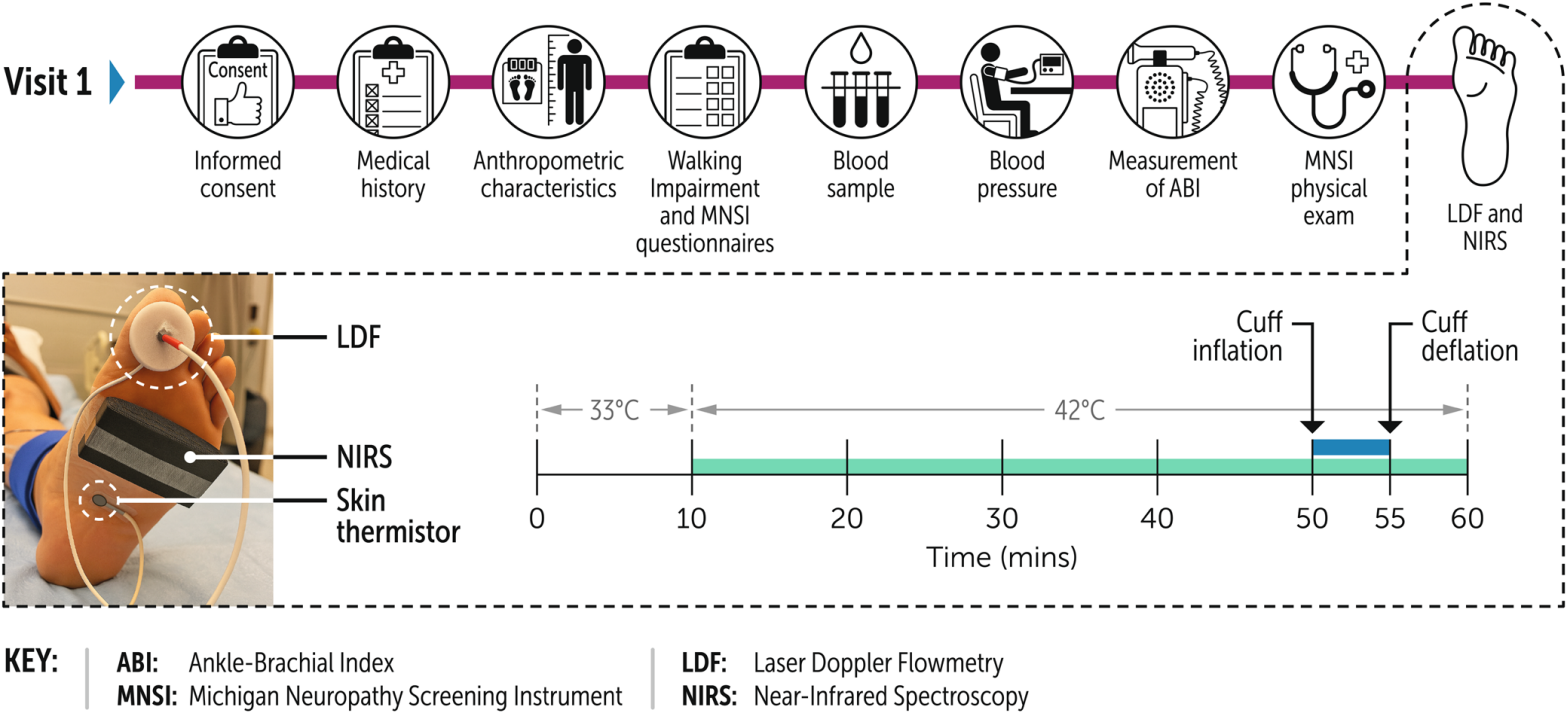
Overview of Visit 1 procedures and experimental protocol. Following written informed consent, participants completed baseline screening procedures, including medical history, anthropometric measurements, administration of the Walking Impairment and Michigan Neuropathy Screening Instrument (MNSI) questionnaires, fasting blood sampling, blood pressure measurement, ankle–brachial index (ABI) assessment, and MNSI physical examination. After completion of screening, participants were positioned in a semi‐recumbent posture and instrumented for simultaneous assessment of plantar skin temperature, plantar hallux cutaneous blood flow using laser Doppler flowmetry (LDF), and plantar forefoot tissue oxygenation using frequency‐domain near‐infrared spectroscopy (FD‐NIRS).

Participants completed two morning experimental sessions separated by at least 48 h but no more than 7 days to minimize potential temporal and clinical variability between visits. Prior to each visit, they were instructed to fast for at least 8 h, avoid structured exercise for 24 h, and refrain from smoking for at least 4 h. Usual morning medications were permitted. The procedures for Visit 1 are summarized in Figure 1. Upon arrival, written informed consent was obtained, followed by baseline screening that included a detailed medical history, anthropometric measurements, administration of the Walking Impairment Questionnaire, a fasting blood draw for glucose and HbA1c assessment, and measurement of resting blood pressure using an automated oscillometric device (Tango+, SunTech Medical, Morrisville, NC). Thermal sensation in the feet and calves was evaluated using a heated electrical pad. Participants then underwent a resting lower‐extremity arterial examination to determine the ankle–brachial index (ABI) and a comprehensive foot sensory assessment using the Michigan Neuropathy Screening Instrument (MNSI).

Following screening, participants rested in a semi‐recumbent position and were instrumented for simultaneous bilateral assessment of foot skin temperature, plantar hallux cutaneous blood flow, and plantar forefoot tissue oxygenation. Skin temperature was measured on both feet using thermocouples (MLT422; ADInstruments, Colorado Springs, CO) secured to the dorsal and plantar surfaces. Cutaneous red blood cell flux was assessed using single‐point laser Doppler flowmetry probes housed within temperature‐controlled heating modules (Moor Instruments, Axminster, United Kingdom). Plantar forefoot tissue oxygenation was measured using a frequency‐domain, dual‐channel near‐infrared spectroscopy system (OxiplexTS; ISS Inc., Champaign, IL), with optical probes secured using medical adhesive and covered with black elastic wraps. Both feet were additionally draped with a dark cloth to further minimize ambient light contamination.

The protocol began with a 10‐min baseline period during which heating modules were maintained at 33°C. Skin temperature at both halluces was then increased at a rate of 0.1°C s^−1^ to 42°C and maintained for 50 min. After 40 min of heating at 42°C, bilateral ankle cuffs were inflated to 220 mmHg for 5 min to induce arterial occlusion. Cuffs were subsequently released, and reperfusion responses were recorded for an additional 5 min. Systolic and diastolic blood pressures were measured at 5‐min intervals throughout the protocol using an automated oscillometric device (Tango+, SunTech Medical, Morrisville, NC).

**FIGURE 2:**
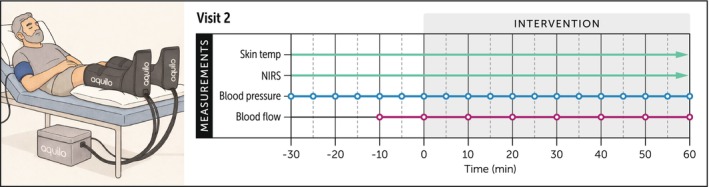
Schematic of Visit 2 experimental protocol. Participants completed a within‐subject intervention in which one leg received combined local HT and low‐pressure IPC, while the contralateral leg served as a control. Skin temperature and plantar forefoot tissue oxygenation (StO_2_; NIRS) were measured continuously throughout the protocol. Blood pressure was obtained at 5‐min intervals, and popliteal artery diameter and blood velocity (for calculation of blood flow) were measured bilaterally at discrete time points during baseline and throughout the 60‐min intervention. The shaded region denotes the intervention period. IPC, intermittent pneumatic compression; NIRS, near‐infrared spectroscopy.

Participants returned to the laboratory on a separate day to evaluate the acute physiological responses to a 60‐min session of localized HT combined with low‐pressure IPC. The limb with the lower ABI received the intervention, whereas the contralateral limb served as an internal control. A schematic of the Visit 2 protocol is provided in Figure 2. As in Visit 1, thermocouples and NIRS probes were positioned bilaterally to continuously measure foot skin temperature and plantar forefoot tissue oxygenation. Black socks were placed over both feet to minimize interference from ambient light. A boot‐style, water‐circulating garment (Aquilo Sports, Louisville, KY) was fitted to the treatment limb and connected to a control unit housing the water reservoir, heater, and pneumatic and water pumps. An identical boot was placed on the contralateral limb but remained disconnected throughout the protocol. An automated oscillometric blood pressure monitor (Tango+, SunTech Medical, Morrisville, NC) was placed on the left arm for repeated blood pressure measurements at 5‐min intervals. Following instrumentation, participants rested quietly for a 30‐min baseline period. Popliteal artery blood velocity and diameter were measured bilaterally using Doppler ultrasound (Vivid IQ, GE Healthcare, Milwaukee, WI, USA) at 10‐min intervals, beginning after the first 20 min of baseline rest. The intervention then commenced with water heated to 40°C being circulated through the boot on the treatment limb, while pneumatic bladders were inflated to 20 mmHg (2‐s inflation/2‐s deflation) for 60 min.

### 2.4 Experimental Measurements

#### 2.4.1 Blood Pressure

Resting blood pressure was measured using an automated oscillometric device (Tango+, SunTech Medical, USA) in accordance with the American Heart Association's standardized recommendations for office‐based blood pressure assessment [48]. Participants were seated upright in a chair with their feet flat on the floor and their back fully supported. Clothing was removed from the upper arm to ensure proper cuff placement, and participants were instructed not to talk, move, or use electronic devices during the measurement period. The participant rested quietly for 5 min before the first measurement. Three measurements were obtained from each arm at 1‐min intervals. The arm exhibiting the higher average systolic pressure was designated as the reference arm for subsequent assessments. The resting blood pressure was the mean of three consecutive readings from the selected arm.

#### 2.4.2 Ankle–Brachial Index (ABI)

The ABI was assessed according to the American Heart Association's recommendations [49]. Participants rested supine for 5 min before measurements were initiated. Pneumatic cuffs were placed on both upper arms and immediately proximal to each malleolus. Using a 5‐MHz handheld Doppler probe, systolic pressures were measured first using a counterclockwise order (right brachial, right posterior tibial, right dorsalis pedis, left posterior tibial, left dorsalis pedis, left brachial), followed by a second pass in the reverse sequence, yielding duplicate measurements for all sites [49]. The ABI for each leg was calculated by dividing the mean of the dorsalis pedis and posterior tibial systolic pressures by the mean of the four brachial pressures [50]. This method of averaging ankle pressures has been shown to provide superior predictive validity for functional outcomes in PAD compared with alternative approaches, such as selecting the highest or lowest ankle pressure within a leg [50].

#### 2.4.3 The Michigan Neuropathy Screening Instrument (MNSI)

Peripheral neuropathy was evaluated using the MNSI, which includes a symptom questionnaire and a standardized physical exam [51, 52]. The 15‐item questionnaire (score range: 0–13) was completed by the patient. A score of 7 or higher was considered abnormal [51, 52]. The physical exam included inspection of the feet, testing of vibration perception, ankle reflexes, and monofilament testing. Each foot was checked for deformities, calluses, fissures, ulcers, or signs of Charcot arthropathy (1 point per abnormal foot). Vibration sensation was tested at the hallux with a 128‐Hz tuning fork and scored as present (0), reduced (0.5), or absent (1). Achilles tendon reflexes were elicited with a reflex hammer, with reinforcement if needed, and scored as present (0), present with reinforcement (0.5), or absent (1). Light‐touch sensation was tested using the 5.07 Semmes–Weinstein monofilament (McKesson #16‐MT34X). The monofilament was applied to eight plantar sites per foot: the plantar surface of the hallux; the second, third, and fifth toes; the first and fifth metatarsal heads; the mid‐plantar arch; and the plantar heel. At each site, the monofilament was pressed perpendicularly until it bent and held for 1–2 s. With their eyes closed, participants were asked whether they felt the stimulation. Monofilament sensation was scored as present (0), reduced (0.5), or absent (1) for each foot based on the number of correctly identified sites (present: ≥ 7 of 8 sites detected; reduced: 1–6 sites detected; absent: 0 sites detected). The physical exam score ranged from 0 to 8; scores above 2 were considered abnormal.

#### 2.4.4 Cutaneous Vascular Function

Red blood cell flux and local skin temperature signals from the heating modules were acquired continuously at 40 Hz and stored for offline analysis using PowerLab/LabChart (ADInstruments). Cutaneous vascular conductance (CVC) was calculated as laser‐Doppler flux divided by mean arterial pressure and evaluated across four physiologically distinct phases: baseline, initial peak, plateau, and reactive hyperemia (RH). Baseline CVC was defined as the final 2 min of the 10‐min period at 33°C, representing resting vasomotor tone under thermoneutral conditions. The initial peak after heating onset was identified as the highest 30‐s segment during the first 5 min of heating. Sustained vasodilation was assessed during the heating plateau at 42°C, defined as the highest stable 5‐min segment immediately preceding arterial occlusion. RH was elicited by 5 min of arterial occlusion (220 mmHg) and quantified as the highest 30‐s CVC value following cuff release. The maximal CVC value was used to compute normalized conductance, expressed as %CVCmax for all phases. The inclusion of RH is supported by evidence that superimposing arterial occlusion on sustained heating elicits greater vasodilation than either stimulus alone, thereby revealing additional vasodilatory reserve [53].

#### 2.4.5 Forefoot Tissue Oxygenation

Forefoot tissue oxygenation was measured using a frequency‐domain near‐infrared spectroscopy system (FD‐NIRS; OxiplexTS, ISS Inc., Champaign, IL) equipped with an adult flexible optical probe. The flexible probe consists of eight emitters and one detector embedded within a low‐profile polyurethane sensor designed for measurements on curved surfaces. The probe configuration included four emitter‐detector separations (2.0, 2.5, 3.0, and 3.5 cm), with colinear emitter‐detector geometry to maximize overlap of the sampled tissue volume. The OxiplexTS system was calibrated before each experiment using the manufacturer‐supplied calibration block in accordance with manufacturer's recommendations. Probe positioning and signal quality were verified before each acquisition by confirming linearity of the AC, DC, and phase slope relationships across emitter distances, as recommended by the manufacturer.

Before probe placement, the plantar forefoot skin was carefully cleaned using ethanol wipes. The flexible probe was positioned over the plantar forefoot and secured using medical tape and black self‐adherent wrap. During Visit 1, the lower leg and foot were additionally covered with a dark sheet, whereas during Visit 2, the probe and foot were covered with a black sock to further reduce light exposure. Although tissue thickness and callus burden were not quantified, care was taken to position the probe over intact skin with adequate probe contact in all participants.

FD‐NIRS provides absolute concentrations of oxy‐[Hb + Mb], deoxy‐[Hb + Mb], and total‐[Hb + Mb] by resolving tissue absorption and scattering properties. Tissue oxygen saturation (StO_2_, %) was calculated as oxy‐[Hb + Mb] divided by total‐[Hb + Mb]. Data were processed offline to quantify baseline values and the dynamic responses to arterial occlusion and reperfusion. The desaturation slope during occlusion (slope 1) was calculated using linear regression over the first 60 s following cuff inflation, and the reperfusion slope (slope 2) was calculated over the first 10 s after cuff release. We also derived the change from baseline to peak (Δ) and time‐to‐peak (TTP), providing a comprehensive assessment of forefoot oxygenation dynamics and microvascular reactivity [54].

#### 2.4.6 Popliteal Artery Blood Flow

Popliteal artery blood flow was measured simultaneously in both legs using high‐resolution duplex Doppler ultrasound (Vivid IQ, GE Healthcare, Milwaukee, WI, USA). A 9‐MHz linear‐array transducer was positioned over the popliteal artery just distal to the popliteal fossa. Diameter (2D B‐mode) and blood velocity (pulsed‐wave Doppler) were acquired in duplex mode using a 5‐MHz Doppler transducer with an insonation angle of 60°. The sample volume was adjusted to encompass the entire arterial lumen without extending beyond the vessel walls, and the Doppler cursor was placed at mid‐vessel. Recordings were obtained every 10 min beginning after the 20‐min baseline rest period and continuing through the 60‐min intervention. At each time point, two 1‐min clips were collected: (I) a 2D B‐mode recording to quantify arterial diameter and (II) a duplex recording with simultaneous diameter and Doppler velocity signals. Mean blood velocity (*V*mean), calculated as the angle‐corrected, intensity‐weighted mean velocity over the cardiac cycle, was derived using the system's automated software. ECG R‐wave–gated frames were exported for offline analysis using automated edge detection and arterial wall tracking software (Cardiovascular Suite, Quipu, Pisa, Tuscany, Italy). Regions of interest corresponding to the optimal vessel segment were identified and held constant throughout the analysis. Popliteal artery blood flow (mL min^−1^) was calculated as: *V*mean × *π* × (diameter/2)^2^ × 60.

#### 2.4.7 Statistical Analysis

This study was designed as an exploratory investigation to assess the feasibility and responsiveness of the experimental procedures and outcome measures in preparation for a larger, adequately powered study. Accordingly, all analyses should be interpreted as exploratory and hypothesis‐generating rather than confirmatory. All statistical analyses were performed using SAS (SAS Institute, Cary, NC). Data are presented as means ± SD unless otherwise stated, and statistical significance was assessed using a two‐sided *α* level of 0.05. For Visit 1, between‐group comparisons (Control, T2D, PAD) of cutaneous vascular conductance normalized to maximal values (%CVCmax) and FD‐NIRS–derived plantar forefoot StO_2_ were evaluated using two‐way repeated‐measures ANOVA, with group as the between‐subjects factor and time (e.g., baseline, initial peak, plateau) as the within‐subject factor. When significant group × time interactions were observed, simple effects (slice) analyses were performed to assess group differences at each time point, followed by post hoc pairwise comparisons with appropriate adjustment for multiple testing. The rates of deoxygenation and reoxygenation (slopes 1 and 2) were compared between groups using one‐way ANOVA, followed by Tukey‐adjusted post hoc comparisons when appropriate.

For Visit 2, the acute effects of combined HT and IPC were evaluated using a within‐subject design, with one leg receiving treatment and the contralateral leg serving as a control. For each outcome variable, the primary dependent measure was the interlimb difference (treated leg minus control leg; TL − CL), calculated at baseline and after 60 min of treatment. These interlimb difference values were analyzed using a two‐way repeated‐measures ANOVA with group (Control, T2D, PAD) as the between‐subject factor and time (baseline vs. 60 min) as the within‐subject factor. When significant group × time interactions were detected, slice analyses were performed, followed by Bonferroni‐adjusted pairwise comparisons.

## 3 Results

### 3.1 Subject Characteristics

Table 1 summarizes the demographic and clinical characteristics of the 40 participants enrolled in the study. Between‐group differences were assessed using one‐way ANOVA with Tukey's post hoc testing. Age did not differ significantly among groups (*F* = 1.88, *p* = 0.17; Control: 63.2 ± 8.4 years; T2D: 65.2 ± 7.8 years; PAD: 69.9 ± 9.3 years). Body weight differed among groups (*F* = 4.44, *p* = 0.019), with post hoc analysis indicating higher body weight in the T2D group compared with controls, whereas values in the PAD group did not differ significantly from either group. In contrast, height (*F* = 1.08, *p* = 0.35) and BMI (*F* = 2.26, *p* = 0.12) did not differ significantly among groups. Glycemic control differed significantly among groups for both HbA1c (*F* = 13.31, *p* < 0.0001) and fasting blood glucose (*F* = 13.99, *p* < 0.0001). Post hoc analyses indicated higher values in both the T2D and PAD groups compared with controls, with no difference between T2D and PAD. Notably, 6 of the 9 participants in the PAD group met the diagnostic criterion for T2D (HbA1c ≥ 6.5%).

**TABLE 1:** Participant demographic, clinical, and baseline characteristics.

	Control (*n* = 20)	T2D (*n* = 11)	PAD (*n* = 9)
Demographic characteristics			
Age, mean (SD), year	63.2 (8.4)	65.1 (7.8)	69.8 (9.3)
Sex, *n* (%)			
Male	4 (20)	5 (45.4)	7 (77.8)
Female	16 (80)	6 (54.6)	2 (22.2)
Race and ethnicity, *n* (%)			
White	18 (90)	11 (100)	8 (88.9)
African American or Black	1 (5)	—	—
Asian	1 (5)	—	—
American Indian or Alaska Native	—	—	1 (11.1)
Weight, mean (SD), kg	76.3 (14.6)	92.8 (14.2)^a^	89.5 (21.0)
Height, mean (SD), cm	168.8 (10.4)	174.2 (11.4)	173.0 (10.1)
Body Mass Index, mean (SD), kg/m^2^	26.8 (5.0)	30.5 (4.2)	29.7 (6.0)
Systolic blood pressure, mean (SD), mmHg	135.2 (13.8)	139.4 (11.5)	140.4 (18.1)
Diastolic blood pressure, mean (SD), mmHg	82.6 (11.8)	80.5 (10.4)	75.7 (9.9)
Hemoglobin A1c, mean (SD), %	5.8 (0.4)	7.4 (1.0)^a^	7.0 (1.3)^a^
Fasting blood glucose, mean (SD), mg/dL	101.9 (19.2)	157.8 (36.3)^a^	151.3 (46.2)^a^
Ankle–Brachial Index (ABI)	1.13 (0.06)	1.14 (0.17)	0.64 (0.13)^ab^
Smoking status			
Previously smoked cigarettes	4 (20)	3 (27.3)	6 (66.7)
Currently smoking cigarettes	1 (5)	1 (9.1)	2 (22.2)
Medication use			
Antidiabetic agents	4 (20)	11 (100)	7 (77.8)
Statins	7 (35)	7 (63.6)	8 (88.9)
Non‐statin lipid‐lowering agents	—	1 (9.1)	1 (11.1)
Antiplatelet agents	2 (10)	4 (36.3)	7 (77.8)
Anticoagulants	—	2 (18.2)	3 (33.3)
ACE inhibitors/ARBs	6 (30)	5 (45.5)	6 (66.7)
Calcium channel blockers	4 (20)	2 (18.2)	3 (33.3)
Beta‐blockers	2 (10)	4 (36.3)	7 (77.8)
Diuretics	1 (5)	4 (36.3)	2 (22.2)
Nitrates/Vasodilators	—	2 (18.2)	1 (11.1)
Walking impairment (WIQ)			
WIQ distance score	95.6 (8.0)	78.5 (30.8)	55.6 (17.8)^ab^
WIQ speed score	85.6 (17.0)	64.7 (37.4)	39.5 (18.2)^ab^
WIQ stairs score	89.1 (15.3)	72.2 (30.4)	44.7 (23.9)^ab^
Peripheral neuropathy (MNSI)			
MNSI—Questionnaire	1.3 (2.0)	3.5 (2.7)	5.3 (1.5)^a^
MNSI—Physical examination	1.6 (1.5)	1.8 (1.6)	3.1 (2.3)

*Note:* Values are presented as mean ± SD for continuous variables and *n* (%) for categorical variables. Participants were categorized as non‐diabetic controls (Control; *n* = 20), individuals with type 2 diabetes (T2D; *n* = 11), or individuals with peripheral artery disease (PAD; *n* = 9), with or without T2D. Between‐group differences were assessed using one‐way ANOVA for continuous variables, followed by Tukey's post hoc test when appropriate. Values marked with a indicate ^a^ significant difference vs. Control (*p* < 0.05), and values marked with ^b^ indicate a significant difference vs. T2D (*p* < 0.05); values marked with ^ab^ indicate a significant difference vs. both Control and T2D.

Abbreviations: ABI, ankle–brachial index; ACE, angiotensin‐converting enzyme; ARB, angiotensin receptor blocker; MNSI, Michigan Neuropathy Screening Instrument; WIQ, Walking Impairment Questionnaire.

Blood pressure did not differ among groups for either systolic (*F* = 0.67, *p* = 0.52) or diastolic (*F* = 0.67, *p* = 0.52) pressure. Patient‐reported peripheral neuropathy symptoms, as assessed using the MNSI questionnaire, differed among groups (*F* = 11.02, *p* = 0.0002), with higher scores in PAD than in controls, whereas T2D values were intermediate and not different from either group. In contrast, MNSI examination scores did not differ significantly among groups (*F* = 1.77, *p* = 0.18). The ABI differed markedly among groups (*F* = 59.00, *p* < 0.0001), with lower values in PAD compared with both controls and T2D, whereas controls and T2D did not differ. Walking impairment, assessed by the WIQ, was reduced in PAD across all domains, including distance (*F* = 16.17, *p* < 0.0001), speed (*F* = 12.84, *p* < 0.0001), and stair climbing (*F* = 11.19, *p* = 0.0002). Post hoc analyses indicated lower PAD scores compared with both controls and T2D, whereas controls and T2D did not differ. In addition, responses to the WIQ symptom questionnaire indicated that 8 of the 9 participants with PAD reported pain, aching, or cramps in the calves, buttocks, or thighs in at least one leg during walking activities, consistent with intermittent claudication symptoms.

### 3.2 Cutaneous Blood Flow in the Plantar Hallux

**FIGURE 3:**
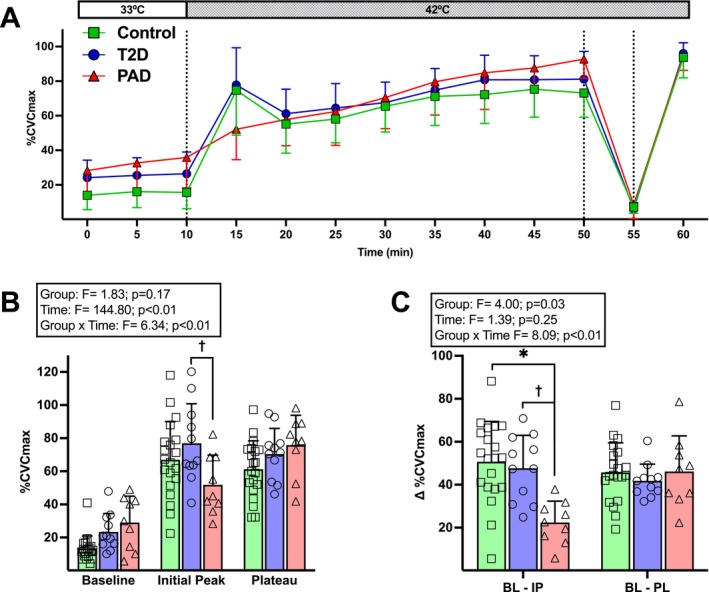
Cutaneous vascular conductance responses to local heating in the plantar hallux. Panel (A) illustrates the time course of %CVCmax during local heating (33°C to 42°C), ankle cuff inflation, and reactive hyperemia in control participants (green), individuals with type 2 diabetes (T2D; blue), and individuals with peripheral artery disease with and without T2D (PAD; red). Panel (B) summarizes %CVCmax at baseline, initial peak, and plateau. The initial peak was defined as the highest 30‐s segment during the first 5 min of heating, whereas the plateau phase was defined as the highest stable 5‐min segment immediately preceding arterial occlusion. Panel C depicts the changes in %CVCmax from baseline to the initial peak (BL–IP) and from baseline to the plateau phase (BL–PL). Symbols represent individual participants; bars and error bars indicate means ± SD. In panel (B), data were analyzed using two‐way repeated‐measures ANOVA with group as the between‐subject factor and time (baseline, initial peak, plateau) as the within‐subject factor. In panel (C), Δ%CVCmax was analyzed using two‐way repeated‐measures ANOVA with group as the between‐subject factor and time (BL–IP, BL–PL) as the within‐subject factor. Significant group × time interactions were followed by simple effects (slice) analyses with post hoc pairwise comparisons adjusted for multiple testing. *p < 0.05 vs. control; †*p* < 0.05 vs. T2D. *n* = 20 (control), *n* = 11 (T2D), and *n* = 9 (PAD).

Figure 3 presents the time course and summary responses of cutaneous vascular conductance normalized to maximal values (%CVCmax) during local heating, ankle cuff inflation, and reactive hyperemia. The local heating response exhibited the characteristic biphasic pattern observed in the cutaneous circulation, consisting of an initial peak in %CVCmax within the first 5 min of heating, followed by a prolonged secondary plateau reached approximately 20–30 min into heating. A brief transient nadir between the initial peak and plateau phases was evident in controls and T2D, but was largely absent in participants with PAD. At baseline, %CVCmax averaged 13.0% ± 7.6% in controls, 24.1% ± 10.9% in T2D, and 30.1% ± 16.0% in PAD. As shown in panel A, %CVCmax increased in all groups following the transition from 33°C to 42°C, with the response peaking within the first 5 min of heating in controls and T2D. In contrast, participants with PAD exhibited a blunted initial vasodilatory response followed by a more gradual rise in %CVCmax throughout the heating period. In controls and T2D, the initial peak %CVCmax was typically greater than the plateau, whereas in PAD, the plateau exceeded the initial peak in all participants. At the initial peak, %CVCmax averaged 66.9% ± 23.1% in controls, 76.9% ± 23.9% in T2D, and 51.8% ± 17.9% in PAD. During the plateau phase, %CVCmax averaged 61.2% ± 17.1% in controls, 70.4% ± 15.6% in T2D, and 75.8% ± 18.1% in PAD. Maximal CVC was achieved during reactive hyperemia in most participants. In three individuals (one control and two T2D), the initial peak represented the maximal value and was therefore used for normalization. In five participants with PAD, peak CVC occurred during the plateau phase rather than during reactive hyperemia.

Figure 3, panel B summarizes %CVCmax during the baseline, initial peak, and plateau phases identified from the continuous response shown in panel A. The initial peak was defined as the highest 30‐s segment during the first 5 min of heating, whereas the plateau phase was defined as the highest stable 5‐min segment immediately preceding arterial occlusion. A two‐way repeated‐measures ANOVA (group × time) revealed a significant main effect of time (*F* = 144.8, *p* < 0.0001) and a significant group × time interaction (*F* = 6.34, *p* = 0.0002), with no main effect of group (*p* = 0.17). Simple effects (slice) analyses showed no group differences at baseline (*p* = 0.0609) or during the plateau phase (*p* = 0.0910), whereas a group effect was present at the initial peak (*p* = 0.0075). Post hoc pairwise comparisons (Bonferroni‐adjusted) indicated a difference between PAD and T2D at the initial peak (adjusted *p* = 0.0019), with no other pairwise differences reaching statistical significance (Figure 3, panel A).

Figure 3, panel C depicts the changes in %CVCmax from baseline to the initial peak (BL–IP) and from baseline to the plateau phase (BL–PL). A two‐way repeated‐measures ANOVA demonstrated a main effect of group (*F* = 4.00, *p* = 0.0268) and a group × time interaction (*F* = 8.09, *p* = 0.0012), with no main effect of time (*p* = 0.25). Slice analyses indicated a group effect for the change from baseline to the initial peak (*p* = 0.0001), but not for the change to the plateau (*p* = 0.9833). Post hoc comparisons showed differences between PAD and controls (adjusted *p* < 0.0001) and between PAD and T2D (adjusted *p* = 0.0009) for the initial peak response, with no difference between controls and T2D (Figure 3, panel C). No pairwise differences were observed for the change from baseline to the plateau phase.

### 3.3 NIRS‐Derived Microvascular Function in the Plantar Forefoot

**FIGURE 4:**
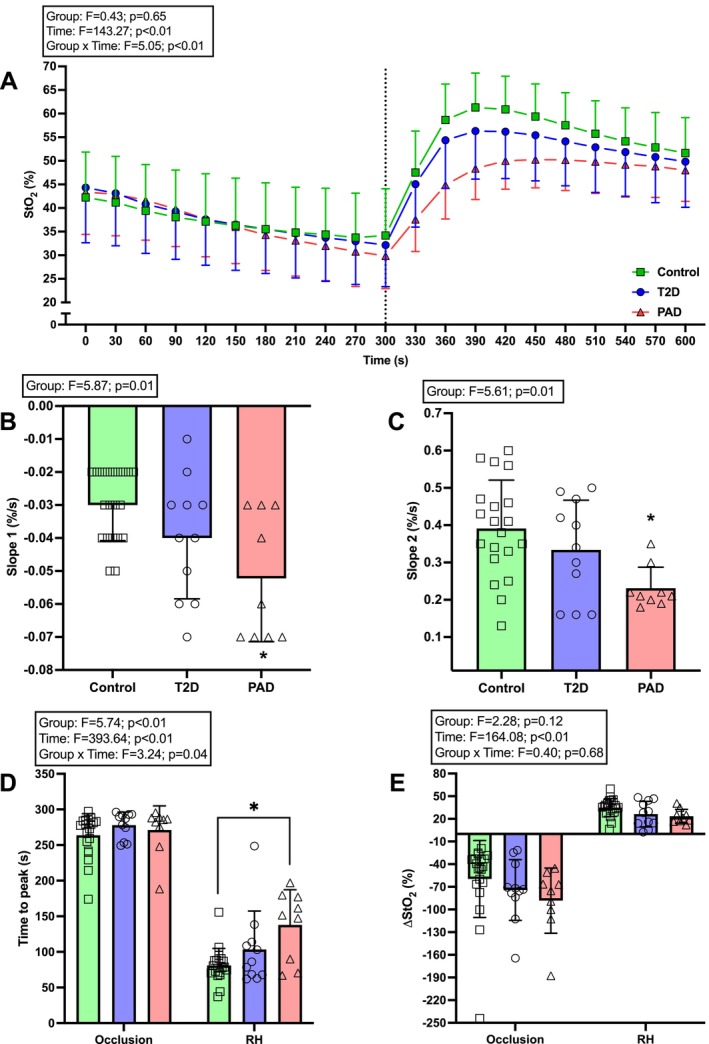
Plantar forefoot tissue oxygenation responses during reactive hyperemia. StO_2_ is shown continuously during baseline, occlusion, and reactive hyperemia (RH) (A), with summary indices including the rate of desaturation during occlusion (slope 1) (B), the rate of reoxygenation during RH (slope 2) (C), time to peak StO_2_ during occlusion and RH (D), and the change in StO_2_ (ΔStO_2_) during occlusion and RH (E) in control participants (green), individuals with type 2 diabetes (T2D; blue), and individuals with peripheral artery disease (PAD; red), including PAD participants with and without T2D. Symbols represent individual participants; bars indicate means ± SD. For (A), StO_2_ responses were analyzed using two‐way repeated‐measures ANOVA with group as the between‐subject factor and time as the within‐subject factor. For slope 1 (B) and slope 2 (C), between‐group differences were assessed using one‐way ANOVA followed by Tukey‐adjusted post hoc comparisons. For (D) and (E), summary outcomes were analyzed using two‐way repeated‐measures ANOVA with group as the between‐subject factor and condition (occlusion, RH) as the within‐subject factor. When significant group × time (or condition) interactions were detected, simple effects (slice) analyses were performed with post hoc pairwise comparisons adjusted for multiple testing. *p < 0.05 vs. control.

Plantar forefoot microvascular oxygenation was assessed using FD‐NIRS (Figure 4). Baseline StO_2_ values were similar across groups (Control: 42.2% ± 9.6%; T2D: 44.3% ± 11.7%; PAD: 43.4% ± 9.0%). During the 5‐min arterial occlusion, StO_2_ declined progressively in all groups, with average reductions of −8.0% ± 4.4% in controls, −12.2% ± 5.8% in T2D, and −13.5% ± 4.7% in PAD. Following cuff release, StO_2_ increased rapidly, reaching peak values approximately 90–120 s after deflation. Peak StO_2_ values averaged 61.6% ± 7.2% in controls, 56.6% ± 10.1% in T2D, and 51.6% ± 5.4% in PAD.

The rate of deoxygenation during occlusion (slope 1; Figure 4B) differed among groups (one‐way ANOVA: *F* = 5.87, *p* = 0.0061), with post hoc comparisons (Tukey‐adjusted) indicating a difference between the control and PAD groups (adjusted *p* = 0.0054). Similarly, the rate of reoxygenation during reactive hyperemia (slope 2; Figure 4C) differed among groups (*F* = 5.61, *p* = 0.0075), with post hoc comparisons identifying a difference between the control and PAD groups (adjusted *p* = 0.0053).

Time to peak StO_2_ (Figure 4D) was analyzed using a two‐way repeated‐measures ANOVA (group × time; occlusion vs. reactive hyperemia), revealing a main effect of group (*F* = 5.74, *p* = 0.0048), a main effect of time (*F* = 393.64, *p* < 0.0001), and a significant group × time interaction (*F* = 3.24, *p* = 0.0449). Slice analyses showed no group effect during occlusion (*p* = 0.5471), whereas a group effect was present during reactive hyperemia (*p* = 0.0005). Post hoc pairwise comparisons (Bonferroni‐adjusted) indicated a difference between the control and PAD groups during reactive hyperemia (*p* = 0.0001), while comparisons between control and T2D (*p* = 0.0900) and between T2D and PAD (*p* = 0.0307) were not statistically significant after adjustment.

The change in StO_2_ from baseline to peak response (Figure 4E), including both the nadir during occlusion and the peak during reactive hyperemia, was analyzed using a two‐way repeated‐measures ANOVA (group × time). This analysis showed a main effect of time (*F* = 164.08, *p* < 0.0001), with no main effect of group (*p* = 0.1166) and no group × time interaction (*p* = 0.6755).

### 3.4 Acute Effects of Combined IPC and HT

**FIGURE 5:**
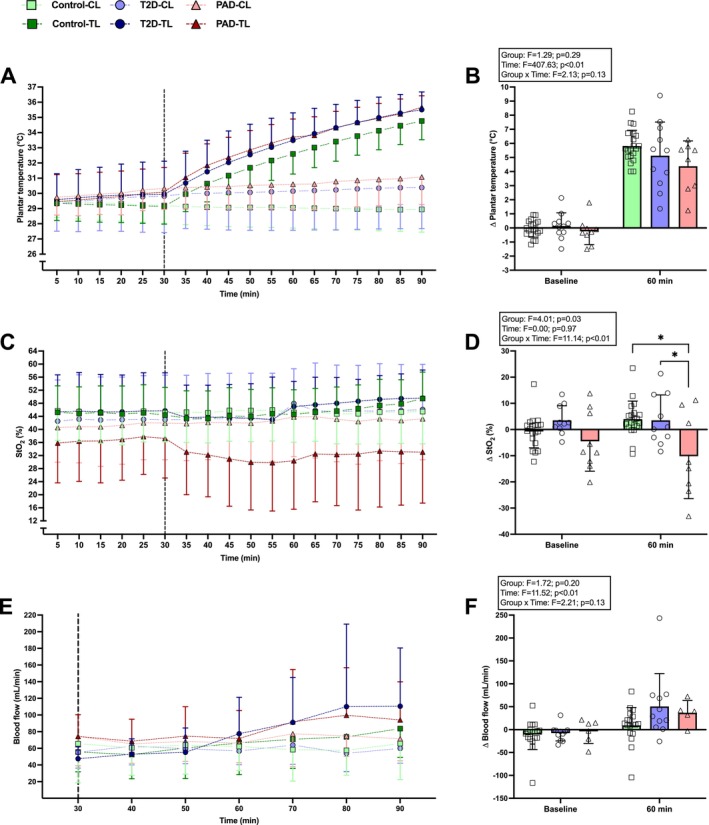
Acute effects of combined heat therapy (HT) and intermittent pneumatic compression (IPC) on plantar temperature, plantar forefoot oxygenation, and popliteal artery blood flow. Acute physiological responses to a boot‐like device delivering combined HT and low‐grade IPC to one leg (treated leg, TL) were assessed using a within‐subject design, with the contralateral leg serving as a control (control leg, CL). Data are shown for control participants (green), individuals with type 2 diabetes (T2D; blue), and individuals with peripheral artery disease with and without T2D (PAD; red). Panel (A) shows the time course of plantar foot temperature during a 30‐min baseline period followed by 60 min of treatment; the dashed vertical line indicates the onset of HT + IPC. Panel (B) shows the interlimb difference in plantar temperature (Δ temperature), calculated as treated leg minus control leg (TL − CL) at baseline and 60 min. Panel (C) shows the time course of plantar forefoot tissue oxygen saturation (StO_2_) measured by FD‐NIRS. Panel (D) shows the interlimb difference in StO_2_ (ΔStO_2_), calculated as TL − CL at baseline and 60 min. Panel (E) shows the time course of popliteal artery blood flow measured by Doppler ultrasound. Panel (F) shows the interlimb difference in blood flow (Δ blood flow), calculated as TL − CL at baseline and 60 min. Positive values indicate higher values in the treated leg than in the control leg. Symbols represent individual participants and bars indicate group means ± SD. For plantar temperature and StO_2_, sample sizes were *n* = 20 (Control), *n* = 11 (T2D), and *n* = 9 (PAD). For popliteal artery blood flow, complete bilateral measurements were available in *n* = 18 controls, *n* = 11 individuals with T2D, and *n* = 6 individuals with PAD. Statistical analyses were performed on the interlimb difference values (TL − CL) shown in panels (B), (D), and (F) using two‐way repeated‐measures ANOVA with group (Control, T2D, PAD) as the between‐subject factor and time (baseline and 60 min) as the within‐subject factor. When a significant group × time interaction was detected, slice analyses were performed, followed by Bonferroni‐adjusted pairwise comparisons. *p < 0.05 for pairwise differences at 60 min (Control vs. PAD and T2D vs. PAD).

The feasibility and acute physiological effects of combined heat therapy (HT) and low‐grade intermittent pneumatic compression (IPC) were evaluated using a within‐subject design in which one leg received the intervention while the contralateral leg served as a control. To quantify treatment effects, responses were expressed as inter‐leg differences and analyzed using two‐way repeated‐measures ANOVA with factors group (Control, T2D, PAD) and time (baseline vs. 60 min). Figure 5 illustrates the temporal responses and corresponding changes in plantar temperature, plantar forefoot tissue oxygen saturation (StO_2_), and popliteal artery blood flow.

For plantar temperature (Δ temperature; Figure 5B), there was a significant main effect of time (*F* = 407.63, *p* < 0.0001), with no main effect of group (*p* = 0.2861) and no group × time interaction (*p* = 0.1333), indicating that the magnitude of heating relative to the contralateral leg increased similarly across all groups. For plantar forefoot StO_2_ (ΔStO_2_; Figure 5D), there was a main effect of group (*F* = 4.01, *p* = 0.0266) and a significant group × time interaction (*F* = 11.14, *p* = 0.0002), with no main effect of time (*p* = 0.9725). Slice analyses revealed a group effect at 60 min (*p* = 0.0017), but not at baseline (*p* = 0.1956). Within‐group comparisons indicated significant changes over time in the Control (*p* < 0.0001) and PAD (*p* = 0.0116) groups, but not in T2D (*p* = 0.9352). Post hoc pairwise comparisons at 60 min (Bonferroni‐adjusted) showed that ΔStO_2_ differed between Control and PAD (*p* = 0.0005) and between T2D and PAD (*p* = 0.004), whereas Control and T2D did not differ (*p* = 0.7102). These findings indicate a differential StO_2_ response to HT + IPC in PAD compared with the other groups. For popliteal artery blood flow (Δ flow; Figure 5F), there was a significant main effect of time (*F* = 11.52, *p* = 0.0019), with no main effect of group (*p* = 0.1965) and no group × time interaction (*p* = 0.1267), indicating a similar increase in bulk limb blood flow across groups during the intervention.

## 4 Discussion

The present exploratory study had two primary objectives. First, we performed a comprehensive assessment of microvascular reactivity in the plantar hallux and forefoot among individuals with T2D and PAD, and age‐matched controls. Importantly, the PAD cohort included individuals with and without coexisting T2D, reflecting the clinical heterogeneity of this population and allowing us to capture the integrated impact of macrovascular disease with or without metabolic dysfunction. During local heating of the plantar hallux, individuals with PAD exhibited an altered temporal profile of the vasodilatory response, characterized by an attenuated initial peak and a preserved plateau phase. In contrast, and contrary to our hypothesis, microvascular responses to local heating were largely preserved in the T2D group, with values comparable to controls across phases. We also examined post‐occlusive changes in NIRS‐derived plantar forefoot oxygenation. While baseline StO_2_ and the overall magnitude of the hyperemic response were similar across groups, individuals with PAD demonstrated altered oxygenation dynamics, including differences in time to peak and in the rates of deoxygenation and reoxygenation compared with controls. In contrast, responses in the T2D group were generally comparable to those observed in controls.

As a secondary objective, we evaluated the feasibility of a boot‐like device combining local HT with gentle IPC. A single treatment session elicited significant increases in bulk leg blood flow, assessed at the popliteal artery, with a similar magnitude and time course across groups. However, plantar forefoot oxygenation responses to the intervention differed by group. In controls and individuals with T2D, StO_2_ increased over time and exceeded baseline values by the end of the intervention, whereas in PAD, StO_2_ remained below baseline throughout the intervention. Collectively, these findings indicate that PAD is associated with abnormalities in plantar microvascular reactivity that may contribute to the heightened susceptibility to foot complications, including diabetic foot ulcers.

### 4.1 Cutaneous Blood Flow in the Plantar Hallux

Despite being a common site for complications in T2D and PAD, including diabetic foot ulcers, the microcirculation of the plantar foot has received comparatively little attention compared with other vascular beds, such as the forearm, leg, or dorsal foot. In non‐glabrous skin, local heating elicits a well‐characterized biphasic hyperemic response, consisting of an initial transient peak largely mediated by neural mechanisms, followed by a brief nadir and a sustained plateau phase driven predominantly by locally produced vasodilatory factors [33]. The magnitude and temporal characteristics of these phases vary substantially across skin regions, reflecting heterogeneity in microvascular control mechanisms [55, 56, 57, 58, 59]. In contrast, the glabrous skin has received far less attention, and the mechanisms governing thermal hyperemia in these regions remain incompletely understood. Metzler‐Wilson and colleagues showed that slow local heating of the palm can evoke an initial peak in cutaneous vascular conductance that exceeds that of the subsequent plateau phase in healthy individuals [58]. In agreement with these observations, we found that the initial peak during local heating of the plantar hallux was slightly greater than the plateau phase in both controls and individuals with T2D. Although the underlying mechanisms remain speculative and were not directly assessed in the present study, this distinctive response pattern in glabrous skin may reflect a greater contribution of arteriovenous anastomoses, differences in nitric oxide dependence, or unique features of neural innervation compared with non‐glabrous skin [33, 58].

Relatively few studies have examined cutaneous blood flow responses to local heating of the plantar foot, but available evidence suggests that thermal hyperemia is markedly attenuated in individuals with T2D and established peripheral neuropathy [22, 60, 61], whereas it is largely preserved in those without neuropathy [61]. For example, Stevens and colleagues reported a paradoxical early decline in plantar hallux blood flow during local heating, along with a reduced peak response, in patients with T2D and neuropathy, a pattern not observed in individuals with diabetes without neuropathy [61]. Importantly, responses to local heating of the dorsal foot were comparable across groups, underscoring pronounced regional heterogeneity in cutaneous microvascular function within the foot [61]. We observed that both the initial peak and plateau phases during local heating were similar between controls and individuals with T2D. These results indicate that in the absence of overt peripheral neuropathy, cutaneous microvascular reactivity to thermal stress in the plantar hallux is preserved in T2D.

The risk of foot ulceration and subsequent amputation is highest in individuals with combined PAD and T2D [8], a vulnerability often attributed to profound microvascular dysfunction [7]. Despite this clinical relevance, mechanistic studies of plantar foot microcirculation in this population remain limited. Prior work in PAD has largely focused on microvascular responses in the leg or dorsal foot [25, 27, 28], with relatively few studies examining the toes, particularly within glabrous plantar skin. For example, Jörneskog and colleagues demonstrated impaired capillary blood cell velocity in the nailfold of the hallux during post‐occlusive reactive hyperemia, particularly in patients with concomitant diabetes [62]. To our knowledge, the present study is among the first to assess cutaneous thermal hyperemia in the plantar hallux in individuals with PAD, the majority of whom also had T2D. A central finding of this study was the attenuation of the initial peak phase during local heating in PAD. In glabrous skin of the hands and feet, arteriovenous anastomoses (AVAs) are highly abundant and play a prominent role in thermoregulatory blood flow responses [33, 34]. These structures may contribute to the rapid increase in skin blood flow at the onset of local heating, although the specific mechanisms underlying the initial peak in glabrous skin remain incompletely defined [33]. It is therefore possible that the blunted initial peak observed in PAD reflects, at least in part, impaired neurovascular signaling or altered regulation of these specialized vascular pathways during the early phase of heating. These findings provide evidence of impaired cutaneous microvascular reactivity in the plantar hallux in PAD, which may contribute to the heightened susceptibility to ulceration at this site. Future studies are warranted to further define regional heterogeneity within the foot, delineate the mechanisms underlying phase‐specific impairments in thermal hyperemia, and determine whether these responses have prognostic value for foot ulcer risk in this vulnerable population.

### 4.2 NIRS‐Derived Microvascular Function in the Plantar Forefoot

We employed frequency‐domain near‐infrared spectroscopy (FD‐NIRS) to quantify plantar foot oxygenation before, during, and after ankle‐level circulatory occlusion. This approach enabled assessment of dynamic changes in tissue oxygenation during ischemia and reactive hyperemia [54]. FD‐NIRS offers important methodological advantages over continuous‐wave NIRS techniques by independently quantifying tissue absorption and scattering, thereby enabling absolute measurements of tissue oxygenation while accounting for dynamic changes in tissue optical properties [63]. This distinction is particularly relevant for vascular occlusion and reactive hyperemia protocols, during which tissue optical properties change dynamically [64]. Importantly, the signal reflects a composite contribution from skin, subcutaneous tissue, vascular compartments, and intrinsic foot musculature, providing an integrated measure of plantar tissue oxygenation.

At baseline, plantar forefoot oxygenation was comparable across control, T2D, and PAD groups. In contrast, dynamic responses to ischemia and reperfusion revealed phase‐specific alterations. The rate of deoxygenation during arterial occlusion (slope 1) differed among groups, with PAD exhibiting a steeper decline compared with controls, suggesting a greater imbalance between oxygen delivery and utilization during ischemia. Similarly, the rate of reoxygenation during reactive hyperemia (slope 2) was altered in PAD, consistent with impaired microvascular oxygen delivery. Our findings in the plantar foot extend prior observations in the calf musculature of patients with PAD, in which NIRS and BOLD MRI studies have demonstrated delayed postischemic reoxygenation and prolonged time‐to‐peak responses during reactive hyperemia [65, 66, 67]. Collectively, our findings indicate that the regulation of plantar tissue oxygenation is altered in individuals with PAD, most of whom also had T2D. These alterations in plantar microvascular oxygenation dynamics may contribute to compromised tissue health in regions of the foot that are particularly vulnerable to ulceration.

### 4.3 Acute Effects of Combined IPC and HT

Local heat has been previously used as an adjunct therapy to accelerate wound healing in patients with DFUs. Early work by Alvarez and colleagues demonstrated that the application of a noncontact thermal device that increased skin temperature to approximately 37°C for 1 h, three times daily over 8–12 weeks, accelerated healing of neuropathic diabetic ulcers [68]. Similarly, Petrofsky and colleagues reported enhanced wound healing following local skin heating to ~37°C using an infrared lamp applied three times per week for 30 min over one month [69]. These benefits were attributed, at least in part, to acute increases in blood flow to the wound and surrounding tissues, as demonstrated using laser Doppler imaging [70]. Building on this body of work, a central objective of the present study was to assess the acute physiological effects of a novel device combining HT with IPC to augment leg blood flow and improve foot oxygenation.

We report that a single session of combined HT and IPC elicited significant increases in popliteal artery blood flow that were comparable across groups. On average, blood flow increased approximately 1.4‐fold in controls, 2.1‐fold in individuals with T2D, and 1.6‐fold in those with PAD. While these increases are smaller than those reported with other heat therapy modalities such as hot‐water immersion, they are directionally consistent. For example, Thomas and colleagues demonstrated that 30 min of waist‐level hot‐water immersion at 42°C resulted in an approximate threefold increase in popliteal artery blood flow in both patients with PAD and healthy controls, accompanied by similar increases in calf muscle tissue oxygenation [38]. Differences in the magnitude of hyperemia between studies likely reflect differences in heating modality, as water immersion provides more efficient conductive heat transfer than water‐circulating garments. Nonetheless, these findings support the concept that the capacity to increase bulk limb blood flow in response to thermal stress is largely preserved in individuals with T2D and PAD.

Despite the comparable changes in bulk leg blood flow, changes in plantar oxygenation assessed by FD‐NIRS followed a clear, group‐specific pattern. In control participants, tissue oxygen saturation initially declined in the treated limb, then progressively increased above baseline toward the end of the intervention. Participants with T2D exhibited a similar early decline but a less consistent late increase in StO_2_. In contrast, individuals with PAD demonstrated a sustained reduction in StO_2_ throughout the intervention, with no evidence of recovery above baseline levels. The physiological basis for the initial decline in StO_2_ observed in most participants remains unclear. Notably, Smith and colleagues previously reported a paradoxical reduction in transcutaneous oxygen tension (TcPO_2_) during plantar skin warming in individuals with T2D and in non‐diabetic controls [71]. This response was specific to the plantar surface, whereas TcPO_2_ increased at the dorsal aspect of the foot [71]. In a subsequent study, the authors confirmed these findings and proposed that the paradoxical decline in TcPO_2_ reflected an increase in epidermal oxygen consumption that exceeded the accompanying rise in oxygen delivery [72]. Although TcPO_2_ and NIRS‐derived StO_2_ interrogate different tissue compartments and physiological processes, a similar mismatch between oxygen delivery and utilization may underlie the early decline in StO_2_ observed in the present study.

Regardless of the mechanisms responsible for the initial response, a key finding of the present study is the divergence in late‐phase plantar oxygenation between groups. Most control participants exhibited an increase in StO_2_ toward the end of the intervention, consistent with enhanced microvascular oxygen delivery. This compensatory response was attenuated in T2D and nearly absent in PAD, despite comparable increases in bulk leg blood flow. The failure to augment plantar oxygenation in PAD likely reflects widespread microvascular dysfunction within the cutaneous and skeletal muscle microcirculation, resulting in an impaired ability to match oxygen delivery to local metabolic demand during thermal stress. These findings highlight an important dissociation between upstream conduit artery flow and downstream tissue oxygenation, underscoring the vulnerability of the plantar microcirculation in PAD.

### 4.4 Limitations

Several aspects of the study design and participant characteristics should be considered when interpreting the current findings. First, the control group was defined as having an HbA1c < 6.5%, resulting in the inclusion of individuals with HbA1c values within the prediabetes range (5.7%–6.4%). This is relevant because accumulating evidence indicates that microvascular dysfunction is already present in prediabetes, including impairments in heat‐induced skin hyperemia [73]. As such, the inclusion of individuals with early metabolic impairment in the control group may have attenuated true differences between groups and led to a conservative estimate of disease‐related microvascular dysfunction.

Second, the PAD group included individuals both with and without concomitant T2D, reflecting the common clinical overlap between these conditions. While this enhances the external validity and clinical relevance of the findings, it limits the ability to attribute observed alterations specifically to PAD. Among people with PAD, those with diabetes present a distinct vascular phenotype, often characterized by more distal, diffuse, and calcified disease, which may differentially affect microvascular function [74, 75, 76]. In addition, PAD classification was based primarily on ABI measurements. Although ABI is the recommended first‐line diagnostic test for PAD, it may not fully reflect distal tissue perfusion, particularly in individuals with diabetes and medial arterial calcification [77]. Importantly, none of the participants in our PAD cohort exhibited abnormally elevated ABI values suggestive of non‐compressible arteries. Nevertheless, complementary assessments of distal perfusion, such as toe‐brachial index, toe pressure, or duplex imaging, were not obtained. As a result, we cannot exclude the possibility that heterogeneity in distal perfusion contributed to the variability in plantar microvascular responses observed within the PAD group.

Third, the burden of peripheral neuropathy appeared heterogeneous across both the T2D and PAD groups, complicating interpretation of the findings and likely contributing to variability in the observed microvascular responses [61]. Abnormal monofilament sensation was present in 3 of 11 participants with T2D and 4 of 9 participants with PAD, while neuropathic pain medications were used by 5 of 11 participants with T2D and 3 of 9 participants with PAD. Furthermore, our assessment of peripheral neuropathy was limited. Although the MNSI is a validated screening tool for peripheral neuropathy [78], we did not perform electrodiagnostic testing, such as nerve conduction studies or electromyography, which remain the reference standards for the diagnosis and phenotyping of peripheral neuropathy [79]. Consequently, the true prevalence, severity, and clinical characteristics of peripheral neuropathy were not fully characterized. Future studies incorporating comprehensive neurological assessments are needed to better define the independent and interacting contributions of diabetes, PAD, and peripheral neuropathy to plantar microvascular dysfunction.

Fourth, medication use varied substantially across groups, reflecting differences in underlying disease burden and clinical management. Participants were permitted to take their usual morning medications before testing, and we cannot rule out the possibility that these treatments influenced the observed vascular responses. At the same time, studying participants under their usual medication regimens enhances the clinical relevance of the findings by reflecting real‐world physiological responses rather than responses under controlled medication withdrawal conditions.

Fifth, the acute HT + IPC intervention employed a within‐subject design in which the leg with the lower ABI received treatment while the contralateral leg served as a control. Although this approach minimized inter‐individual variability and allowed each participant to serve as their own control, interpretation is complicated by interlimb differences in disease severity. In the PAD group, the treated limb exhibited a significantly lower ABI than the contralateral limb (0.57 ± 0.14 vs. 0.72 ± 0.16; mean difference = 0.14, *p* = 0.024), indicating greater disease severity in the treated limb. A smaller but statistically significant interlimb difference was also present in the T2D group (1.22 ± 0.48 vs. 1.27 ± 0.53; mean difference = 0.06, *p* = 0.016). Future studies employing randomized crossover or parallel‐group designs may help further isolate treatment effects from potential influences of interlimb vascular asymmetry.

Finally, the relatively small sample size within subgroups reflects the exploratory, pilot nature of this study and may have limited statistical power to detect more subtle between‐group differences, particularly in the T2D cohort. Taken together, these considerations suggest that the present findings should be interpreted as conservative estimates of plantar microvascular dysfunction. Larger, adequately powered studies are needed to confirm these observations and to more precisely define group differences and underlying mechanisms of microvascular dysfunction in the plantar foot across these clinical populations.

### 4.5 Perspectives

The present study provides novel physiological insight into microvascular function of the plantar foot, a region that is both understudied and disproportionately affected by complications in individuals with T2D and PAD. Distal symmetric polyneuropathy typically presents in the toes and progresses proximally [79], and the plantar surface of the toes and forefoot is the most common site for ulcer development [4, 5]. Despite this clinical reality, most previous studies of lower‐extremity microvascular function have focused on the leg or dorsal foot. Compared with controls, participants with PAD, most of whom also had T2D, exhibited an altered temporal pattern of vasodilation during local heating of the plantar hallux and abnormal dynamics of plantar tissue oxygenation during ischemia and reactive hyperemia. Additional evidence for altered plantar microvascular function was provided by the acute HT + IPC intervention. Although the treatment elicited comparable increases in popliteal artery blood flow across groups, plantar tissue oxygenation increased over time in controls and individuals with T2D but remained below baseline in the PAD group, suggesting a dissociation between upstream conduit artery blood flow and downstream plantar tissue oxygenation. Together, these findings support the presence of altered plantar microvascular regulation in individuals with PAD, including those with and without concomitant T2D, and highlight the need for larger studies with more comprehensive vascular and neurological phenotyping to confirm these findings and define the underlying physiological mechanisms.

## Author Contributions

Conceived and designed research: B.T.R. Performed experiments: J.G., A.M., E.C., G.C., L.B., E.S., B.B.L., C.B., B.R., J.O., L.S.A., E.R., E.H.O., D.H., I.A.F., and B.T.R. Analyzed data: J.G., A.M., E.C., G.C., L.B., E.S., B.B.L., C.B., B.R., J.O., L.S.A., E.R., E.H.O., Q.S., and B.T.R. Interpreted results of experiments: All authors. Prepared figures: J.O., L.S.A., and B.T.R. Drafted manuscript: J.O., L.S.A., and B.T.R. Edited and revised manuscript: All authors.

## Funding

This work was supported by the John Martinson Honors College at Purdue University through a Research Breakthrough Award and by the National Institute on Aging (R01 AG073634‐01A1 to B.T.R.).

## Conflicts of Interest

B.T.R. reports consulting services for SharkNinja unrelated to the present work. The remaining authors declare no conflicts of interest.

## Data Availability

The authors have nothing to report.
